# Polydopamine-Coated Alginate Microgels: Process Optimization and In Vitro Validation

**DOI:** 10.3390/jfb14010002

**Published:** 2022-12-20

**Authors:** Iriczalli Cruz-Maya, Simona Zuppolini, Mauro Zarrelli, Elisabetta Mazzotta, Anna Borriello, Cosimino Malitesta, Vincenzo Guarino

**Affiliations:** 1 Institute for Polymers, Composites and Biomaterials (IPCB), National Research Council of Italy, V.le J.F. Kennedy 54, 80125 Naples, Italy; 2 Laboratory of Analytical Chemistry, Department of Biological and Environmental Sciences and Technologies (Di.S.Te.B.A.), University of Salento, Via Monteroni, 73100 Lecce, Italy

**Keywords:** atomization, core-shell, bioactive coatings, in vitro culture

## Abstract

In the last decade, alginate-based microgels have gained relevant interest as three-dimensional analogues of extracellular matrix, being able to support cell growth and functions. In this study, core-shell microgels were fabricated by self-polymerization of dopamine (DA) molecules under mild oxidation and in situ precipitation of polydopamine (PDA) onto alginate microbeads, processed by electro fluid dynamic atomization. Morphological (optical, SEM) and chemical analyses (ATR-FTIR, XPS) confirmed the presence of PDA macromolecules, distributed onto the microgel surface. Nanoindentation tests also indicated that the PDA coating can influence the biomechanical properties of the microgel surfaces—i.e., σmaxALG = 0.45 mN vs. σmaxALG@PDA = 0.30 mN—thus improving the interface with hMSCs as confirmed by in vitro tests; in particular, protein adsorption and viability tests show a significant increase in adhesion and cell proliferation, strictly related to the presence of PDA. Hence, we concluded that PDA coating contributes to the formation of a friendly interface able to efficiently support cells’ activities. In this perspective, core-shell microgels may be suggested as a novel symmetric 3D model to study in vitro cell interactions.

## 1. Introduction

In the last two decades, research on biomaterials has been focused on the development of three-dimensional (3D) scaffolds to guide the interactions among cells into a simulated in vitro microenvironment able to mimic basic features of the tissue extracellular matrix (ECM). For this purpose, hydrogels have been preferentially used, due to their ability to exchange nutrients and cell metabolites across the 3D network formed by their polymer chains [1]. In this view, microgels—that is chemically or physically crosslinked hydrogels with a round shape and average size on the microscale—were proposed as optimal candidates of ECM-like models, because they are able to provide a fully interconnected filamentar structure with externded surface area, tunable permeability and swelling properties modulable as a function of external microenvironmental stimuli [2].

In the past years, several methodologies were explored for tailored manufacturing of microgels for tissue regeneration [3]. Among them, electro-fluid dynamic atomization (EFDA) is emerging as a versatile, low-cost, and high-throughput technology suitable to design customized devices, with a round shape and micrometric size, able to confine morphological and biochemical signals to support in vitro cell interactions. In comparison with other techniques (i.e., microfluidics, emulsions), EFDA allows the manipulation of organic polymers from a different origin—i.e., synthetic or natural one—by applying high voltage electrical forces to them directly in the solution. An accurate control of the voltage, in combination with other parameters (i.e., flow rate and bath composition), offers the opportunity to customize the morphology of particles, from the micro- to submicro- scale, for the production of a wide pattern of devices with peculiar functional properties [4]. In the last years, different kinds of microgels based on polysaccharides such as chitosan, cellulose, and alginate have been developed by EFDA for a wide range of biomedical applications, from tissue engineering [5,6] to drug delivery [7,8,9].

Among them, recent studies have proposed the use of sodium alginates to fabricate tailor-made microgels via EFDA to study cell behavior under simulated microenvironmental conditions, reproducing healthy [10] or cancer tissues [11]. Indeed, due to their peculiar hydrogel-like behavior, sodium alginates can create physically cross-linked 3D networks able to exhibit outstanding features in terms of mechanical and transport properties. These systems can be really suitable for (i) the immobilization and protection of cells during the encapsulation procedure and (ii) the exchange of oxygen, nutrients, metabolites, and small molecules with the external medium—that is mandatory in supporting cells to survive more longer in a simulated tissue microenvironment.

However, as reported in previous works, sodium alginates can present some limits for in vitro applications, mainly due to low adhesive properties—not comparable with those of other natural biopolymers such as gelatin or chitosan [12,13]—and the tendency to rapidly degrade, just after few weeks in cell culture, thus compromising their chemical and mechanical properties [14].

In order to overcome these drawbacks, an improvement in surface properties of the alginate microgels was proposed through the implementation of a simple methodology based on surface treatment in dilute aqueous dopamine (DA) solution under weakly oxidative conditions. Similar approaches have been previously optimized to introduce more and specific functionalities onto a wide range of different substrates (i.e., metals, glass, and polymers), for potential use in the biomedical, environmental, and energy fields [15,16]. Herein, the polymerization of DA molecules onto the microgel surface was optimized in order to form a thin layer of mussel inspired polydopamine (PDA). This strategy can help to improve the surface adhesion properties of the microgel, thanks to specific functionalities of PDA—i.e., catechol and amine groups—typical of aminoacids such as 3,4-dihydroxy-L-phenylalanine (DOPA) and L-lysine, respectively. This condition concurs to create the physicochemical conditions basically required for efficient biological interfacial recognition [17]. Indeed, PDA macromolecules can also work as spacer to conjugate bioactive molecules onto the alginate surfaces without any manipulation or chemical pre-treatment of the surrounding substrate [18,19].

Therefore, it is proposed to design core-shell microgels—inner core of sodium alginate with a thin shell of PDA—to generate a 3D platform with cell-friendly interfaces able to improve in vitro cell interaction studies. At this stage, process conditions will be set to form a PDA layer with tunable biological and biomechanical properties. Moreover, morphological chemical and biomechanical properties will be investigated to preliminarily validate in vitro interface with hMSC.

## 2. Materials and Methods

### 2.1. Materials

Sodium alginate (SA) from brown algae (≈250 cps), anhydrous calcium chloride (CaCl_2_), dopamine-hydrochloride (DA), 2-amino-2-(hydroxymethyl)-1,3-propanediol (Tris base, buffer), and ethanol were purchased by Sigma Aldrich (Milan, Italy). To prepare the solutions, deionized water was used, while 18 G metallic needles (18 Ga) were purchased by BD, USA for the atomization process.

For in vitro assays, phosphate buffer solution (PBS), sodium dodecyl sulfate (SDS), human mesenchymal stem cells (hMSCs, SCC034), fetal bovine serum (FBS), BCA QuantiPro assay kit, bovine serum albumin (BSA), Cell Counting Kit-8 (CCK-8), Cell Proliferation Kit II (XTT, Roche, Basel, Switzerland), streptomycin, penicillin, and L-glutamine were purchased from Sigma Aldrich (Milan, Italy).

### 2.2. Synthesis of Microgels

#### 2.2.1. Core Fabrication: Alginate Atomization

SA was dissolved in distilled water to obtain a 2% wt/v solution. SA solution was processed via EFDA to obtain micro-sized gels according to the schematic procedure reported in the U.S. Patent [20]. Briefly, the SA solution was placed in a 5 mL syringe connected to a power supply by the needle to apply 20 kV. The feed rate of the syringe pump was optimized at 5.0 mL/h, while distance was set at 15 mm to optimize the size and shape of droplets. Droplets were collected into a CaCl_2_ solution (1.1% *w*/*v*) under magnetic stirring in order to easily trigger an ionotropic crosslinking of alginate in water solutions.

#### 2.2.2. Shell Fabrication: PDA Coating

**Figure 1:**
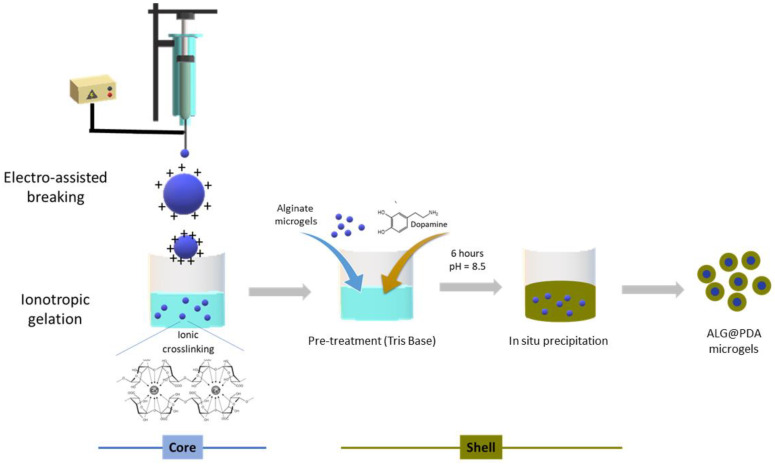
Scheme of fabrication of core-shell microgels.

The alginate microgels were coated with PDA by the following simple procedure: The alginate ALG microgels (about 60 mg) were immersed and dispersed into 10 mL of Tris base solution (10 mM, pH 8.5), and then DA (0.5 or 1.0 mg/mL) was added. The mixture was gently stirred at room temperature for 6 h. The PDA-coated microgels (ALG@PDA) obtained were rinsed and stably stocked in water (Figure 1).

### 2.3. Characterization of Microgels

#### 2.3.1. Morphological Analysis

The morphology of alginate microgels and PDA-coated microgels was evaluated by optical microscopy (DM750, Leica, Wetzlar, Germany). Optical images were used to quantitatively estimate microgel sizes via image analysis (Image J, 1.47; NIH, Bethesda, Rockville, MD, USA). Results were reported as mean ± standard deviation (SD). The surface morphology of samples was investigated by images recorded by Scanning Electron Microscopy (SEM, Quanta FEG 200 FEI, Eindhoven, The Netherlands) working at low-voltage electron emission (2 kV) into a low vacuum range (e.g., chamber pressure < 10^−2^ Pa) to avoid any sample pre-treatment via conductive metal deposition.

#### 2.3.2. Chemical-Physical Characterization

Attenuated total reflectance-Fourier transform infrared (ATR-FTIR) spectra of dried samples were recorded on a Perkin Elmer Spectrum 100 FTIR spectrophotometer (Milano, Italy) in the range of 4000 to 400 cm^−1^ with a resolution of 4 cm^−1^ and 32 scans.

X-ray photoelectron spectroscopy (XPS) measurements were recorded with an AXIS ULTRA DLD (Kratos Analytical, Stretford, UK) photoelectron spectrometer using a monochromatic AlKα source (1486.6 eV) operated at 150 W (10 kV, 15 mA). The base pressure in the analysis chamber was 5.3 × 10^−9^ torr. Survey scan spectra were recorded using a pass energy of 160 eV and a 1 eV step. High resolution spectra were acquired using a pass energy of 20 eV and a 0.1 eV step. In each case, the area of analysis was about 700 μm × 300 μm. During the data acquisition, a system of neutralization of the charge has been used. Processing of the spectra was accomplished by CasaXPS Release 2.3.16 software. The binding energy (BE) scale was referenced to the Au 4f7/2 peak at 84.0 eV. Surface charging was corrected considering adventitious C 1s (BE = 285 eV).

Thermal stability of dried samples was investigated by thermogravimetric analysis (TGA) using a Q500 system by TA Instruments (New Castle, Germany) under N2 atmosphere (50 mL/min) and a heating ramp of 10 °C/min. Sample weights of around 6 ± 0.5 mg were used for the run test performed by heating from ambient temperature to 800 °C.

Swelling tests were performed on dried, uncoated, and PDA-coated alginate microgels. To qualitatively estimate the swelling behavior of ALG and ALG@PDA microgels, swelling tests were performed on dried samples. Microgels were placed onto glass slides in bi-distilled water. Optical images were taken to monitor shape and size changes in the microgels over time.

The nanoindentation technique was employed to assess the extension of dried PDA-coated particles by comparing the attained results with corresponding alginate particles. A Nano Test Platform (Micro Materials Ltd., Wrexham, UK) was used to measure the force-displacement profile in a very tiny range by a controlled force mode, and the force vs. indentation curves were analyzed assuming a different penetration behavior at particles interface. Each test was performed at a loading ramp of 1 mN/min using a three-sided Berkovich pyramidal diamond tip (100 µm radius) up to a maximum penetration depth of 500 nm.

### 2.4. In Vitro Studies

#### 2.4.1. Protein Adsorption

The protein adsorption was analyzed using a BCA protein assay kit (QuantiPro) to evaluate the effect of PDA coating on the absorption of BSA as a model. The microgels (20 mg/mL) were put in a 96-well plate and incubated in 1 mg/mL of BSA solution at 37 °C. After 4 and 24 h the microgels were removed from the protein solution and rinsed three times with PBS. Then, samples were incubated with 1% of SDS solution for 1 h to extract the protein adsorbed by the samples. The protein concentration was detected using a BCA protein assay kit as indicated in the manufacturer’s instructions.

#### 2.4.2. Cell Culture

For in vitro assays, hMSCs were cultured in a 75 cm^2^ cell culture flask in Eagle’s alpha minimum essential medium (α-MEM) supplemented with 10% FBS, antibiotic solution (streptomycin 100 µg/mL and penicillin 100 U/mL), and 2 mM of L-glutamine, incubated at 37° C in a humidified atmosphere with 5% CO_2_ and 95% air. hMSCs from 4 passages were used for cell proliferation assays.

#### 2.4.3. Cell Adhesion and Proliferation

Before the in vitro studies, ALG and ALG@PDA microgels were washed and sterilized in ethanol (70%) for 30 min, then washed three times with PBS. Afterward, ALG and ALG@PDA were placed in a 96-cell culture plate (20 mg/mL) and incubated with cell culture medium. After 30 min, the cell culture media was removed and hMSCs were seeded at 5 × 10^4^ cells/well to perform cell adhesion and proliferation assays. For cell adhesion, after 24 h, the medium was removed, and samples were washed with PBS to remove the unattached cells; then, 100 µL of fresh medium with 10 µL of CCK-8 was added. After 4 h incubation in standard conditions, the supernatant was collected and placed into a microplate reader to measure the absorbance at 450 nm. Results were presented as percentage of cell adhesion with respect to the cell culture plate.

Confocal microscopy was used to observe the interaction of cells with the ALG and ALG@PDA surfaces. hMSCs were preliminarily incubated in phenol red-free medium with CellTracker Deep Red (Thermo Fisher scientific, Waltham, MA, USA) at 37 °C for 30 min. Then, the cell culture was removed, and cells were washed with PBS, and fresh culture media was added to incubate cells for 1 h. Lastly, cells were trypsinized, placed onto the microgels, and incubated in standard conditions for three days. After this period, the samples were washed with PBS and fixed with 4% paraformaldehyde. Then, samples were washed with PBS to evaluate the cell morphology via confocal microscopy (LSM510, Carl Zeiss, Jena, Germany).

For cell proliferation, an XTT assay kit was performed after 1, 3, 7, and 14 days. Briefly, at each time point, the cell culture media was removed and changed by 100 µL of fresh medium with XTT working solution as indicated by the manufacturer’s instructions and incubated for 4 h in standard conditions. The supernatant was collected, and absorbance measured at 450 nm using a microplate reader. Results are presented as mean ± standard error (n = 3). Analysis of variance (ANOVA) with Tukey’s post hoc test was used to detect differences between groups. A value of *p* < 0.05 was considered to determine statistically significant differences.

## 3. Results

Alginates are very attractive biopolymers that are growing in interest in the biomedical field, due to their unique capability to combine well-known properties of biocompatibility with the ease of being processed in different forms (i.e., injectable gels, microbeads, and porous scaffolds) [21]. In particular, spherical-shaped alginate gels can be simply created by dropping a sodium alginate solution into an aqueous bath with divalent cations (i.e., Ca^2+^, Cu^2+^, Mg^2+^, and Sr^2+^) to form stable chain agglomerates easily assembled into 3D networks by physical links mediated interaction of the charged polymer and cation species. This makes the alginate beads particularly suitable to support in vitro cell interactions for different biological and/or therapeutic approaches (i.e., cell encapsulation [22], cell covering [23], and cell confinement into the scaffolds [24]). Recent advances in process technologies enabled the overcoming of some intrinsic limitations in the control of the beads’ size, by introducing the use of external electrical forces to break the polymer solution into smaller droplets, thus promoting the formation of beads on the micrometric scale from tens to hundreds μm [25]. However, drawbacks related to the tendency of alginate microgels to easily degrade in vitro cannot be solved only by an accurate control of the process conditions; it is required that chemical and/or physical signals be included to improve some shortcomings in terms of chemical and/or mechanical properties [26].

This becomes particularly relevant for in vitro studies where the presence of monovalent cations such as Na^+^ tends to substitute bivalent ones (i.e., Ca^2+^) into the alginate network, thus determining a crosslink breaking and, then, a gradual loss of the gel stability in an aqueous solution. Consequently, the mechanical properties of microgels progressively decay in three–four weeks until the complete dissolution of the alginate chain network.

**Figure 2:**
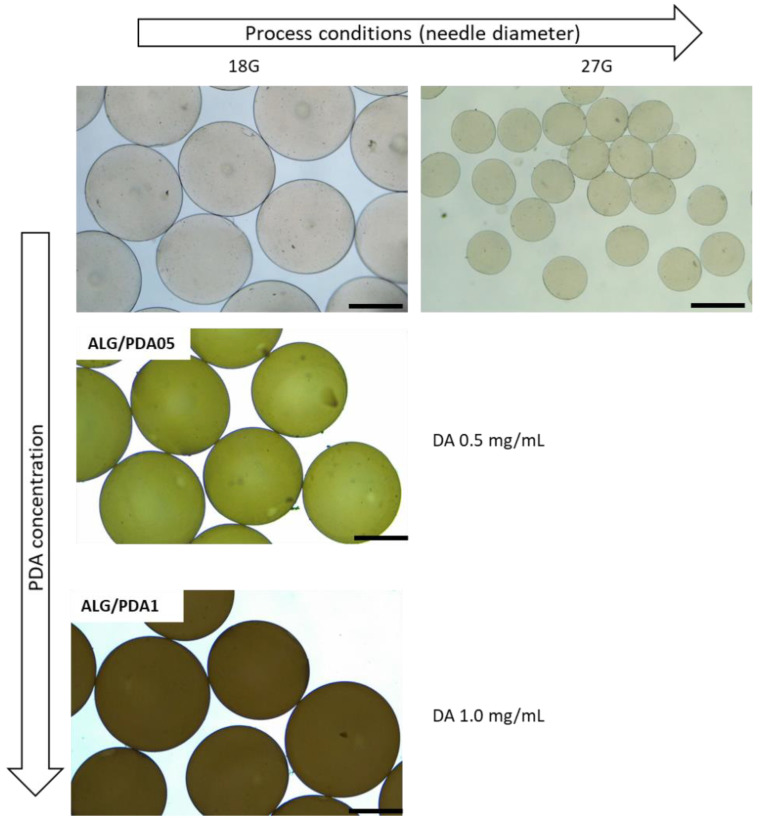
Optical images of alginate microgels (diameter change by the use of different needle diameter, 18 and 27 G), and PDA-coated alginate microgels obtained with different initial DA concentrations: 0.5 mg/mL (ALG@PDA05) and 1.0 mg/mL (ALG@PDA1). (Mag: 4×; Scale bar: 500 µm).

In order to overcome this problem, a simple solution is proposed, by coating alginate microgels fabricated via EFDA with a thin shell of PDA. In our previous work, we have demonstrated that the use of PDA coatings can modify surface properties of porous nanostructured scaffolds to improve cell adhesion and proliferation [27,28]. Herein, the proposed strategy offers the opportunity to form core-shell systems—i.e., alginate core-PDA shell—in a single step at room or body temperature, that may be also compatible with the use of live cells. Indeed, alginate microgels were simply immersed into a 10 mM Tris base solution at a pH = 8.5, and DA was added. The weakly alkaline conditions of the mixture promote the self-polymerization of the DA monomer to PDA, which is spontaneously deposited onto alginate microgels [29]. In this study, two different initial concentrations of DA in Tris-buffer solution were used—i.e., 0.5 and 1.0 mg/mL—leading to PDA-coated alginate microgels named ALG@PDA05 and ALG@PDA1, respectively. Although the mechanism of PDA formation is still a challenging discussion, it was established that a high concentration of DA could increase the formation of some small PDA particles through self-polymerization in solution [29]. Hence, DA concentrations ≤1 mg/mL were recognized to ensure a PDA thin film deposition on ALG surfaces inhibiting polymer aggregate impurities. The morphology of different microgel types was analysed via optical microscopy, and some images are reported in Figure 2. The average diameters of alginate microgels—calculated by image analysis—may significantly vary from 882.75 ± 31.7 to 401.33 ± 29.01 µm by changing the diameter of the needle—18 G or 27 G—fixed to the ejection head, confirmed to play a relevant role in the mechanism of fluid dynamic breaking of the droplet in the presence of electric forces.

Moreover, an evident color change of alginate microgels from clear to dark brown was noted after the PDA coating, prepared with higher DA concentration (1.0 mg/mL). However, no significant changes in the average diameter were detected after the PDA coating deposition, independently of the DA concentration (Table 1).

**Figure 3:**
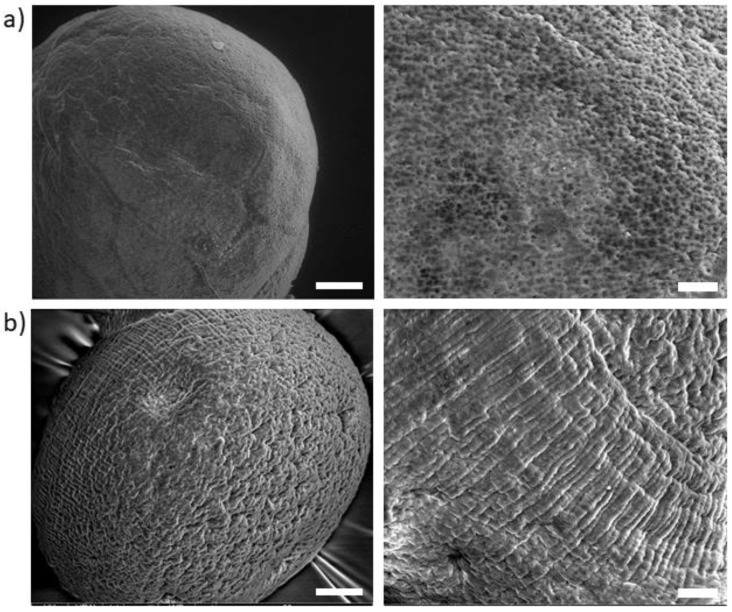
Morphological analysis via low vacuum SEM: evaluation of the surface morphology of (**a**) ALG and, (**b**) ALG@PDA. Scale bar: 20 µm (**left**) and 10 µm (**right**).

Further investigation of the morphological properties of PDA coatings was assessed by SEM analysis performed in low vacuum mode to eliminate any image artifact due to the use of conductive metal coating on the sample surface (Figure 3). In the case of ALG (Figure 3a), a highly porous surface was observed while pores and voids seemed to be completely covered after PDA treatment (Figure 3b). As remarked in highly magnified images, the surface of ALG@PDA clearly shows the presence of a thin and homogeneous coating. Although the mechanism of growth and PDA morphology characteristics are still unclear [30], this result suggests that a highly permeable and hydrophilic surface such that of alginates promotes a more homogeneous deposition of the PDA coating, with respect to a dot-like deposition typically observed on hydrophobic substrates [28,31].

**Figure 4:**
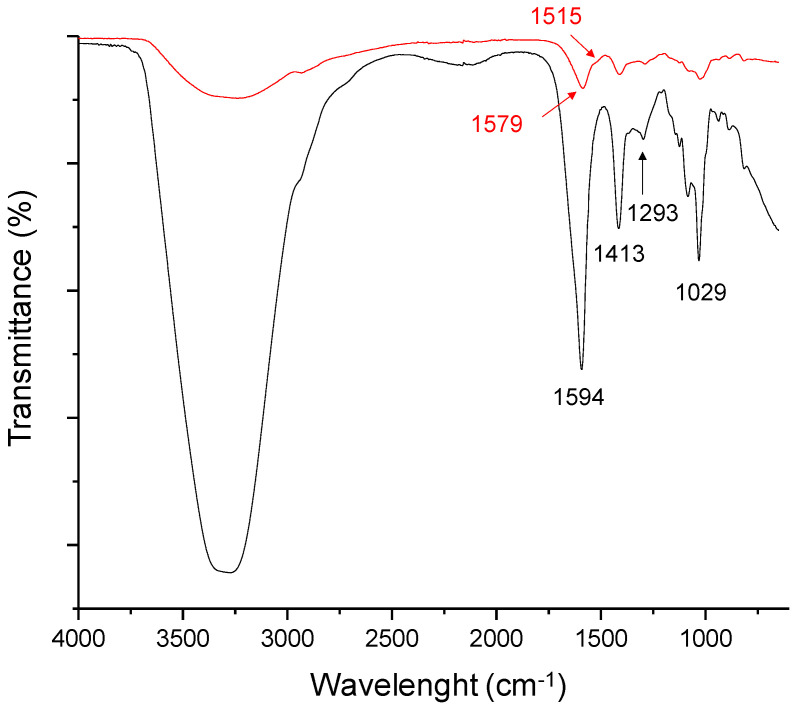
ATR-FTIR spectra of ALG (black curve) and ALG@PDA (red curve). (For interpretation of the references to color in this figure legend, the reader is referred to the Web version of this article.)

The presence of the PDA coating was enhanced by chemical investigations via ATR-FTIR. Figure 4 showed ATR-FTIR spectra of PDA-coated particles (ALG@PDA) in comparison with neat alginate ones (ALG). In the case of the ALG spectrum (black curve), stretching vibrations of O–H bonds of alginate appear in the range of 3600–3000 cm^−1^ as a narrow band, while stretching of aliphatic C–H are weakly observable in the 2940–2840 cm^−1^ range. In addition, the characteristic peaks due to -COOH and C–O–C stretching are observable at 1594 and 1029 cm^−1^, respectively. After PDA deposition, the spectrum (red curve) is very similar to that of alginate, presenting a slight shift of some characteristic peaks (1594–1029 cm^−1^ range) to a lower wavelength, indicating the weakened hydrogen bond network in ALG, due to the presence of the PDA coating. The absorption region (3600–3100 cm^−1^) of stretching vibrations of O-H bonds in ALG@PDA appears broader than in the ALG case. This could be attributed both to additional O–H groups of PDA and key absorption of catechol groups which are included in the same range. The stretching of indole C=C is enhanced by a slight peak at 1515 cm^−1^. Hence, the polymeric shell characteristic peaks show a lower relative intensity than those of uncoated alginate particles, indicating a small core–shell ratio in the formation of ALG@PDA.

**Figure 5:**
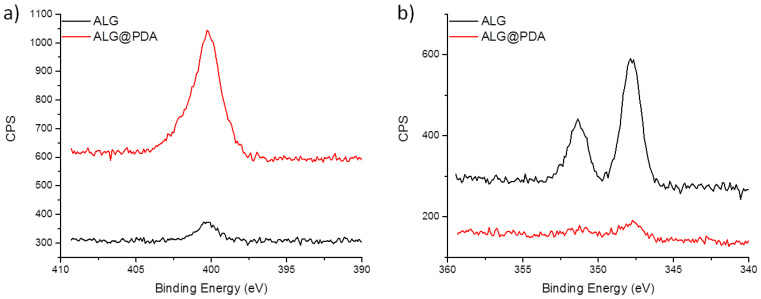
Detailed XPS N 1s (**a**) and Ca 2p (**b**) signals of ALG and ALG@PDA samples. Spectra are charging corrected.

The presence of the PDA coating was further confirmed by XPS analysis performed on both ALG and ALG@PDA particles. Figure 5a reports the comparison on N 1s signal recorded on both samples and evidently shows a remarkable increase on PDA-coated microgels, due to the PDA coating layer. The recorded N 1s on ALG@PDA is centered at about 400 eV and is ascribable to the aminic functionalities of PDA [32]. Interestingly, the presence of the PDA coating on ALG microgels is also evidenced by the significant decrease in Ca 2p signal (Figure 5b) due to its attenuation upon PDA deposition.

**Figure 6:**
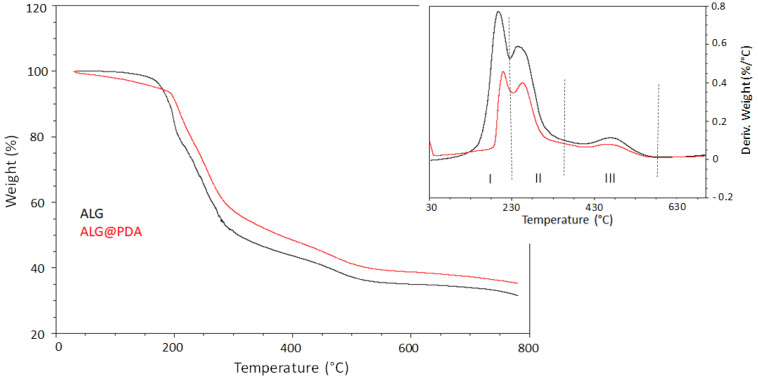
TGA analysis (in N2) of ALG (black curve) and ALG@PDA (red curve) samples. (For interpretation of the references to color in this figure legend, the reader is referred to the Web version of this article).

In order to evaluate the contribution of PDA on the structural properties of ALG and ALG@PDA particles, thermal analyses were also assessed (see all the data summarized in Table 2). The TG spectra (Figure 6) showed similar graph profiles with a good thermal stability for both samples. On the basis of the three main degradation mechanisms enhanced in differential thermogravimetry (DTG) curves (top right graph), in both thermograms the following temperature regions can be identified: the first range from 30–210 °C, the middle range from 210 °C to 300 °C, and the last region temperature above 300 °C. In the ALG spectrum (black curve), the first region showed a low initial weight loss of alginate caused by dehydration, followed by fast degradation to CaCO_3_ with a midpoint of thermal degradation at 197.2 °C (Table 2). In the second temperature region (210–300 °C range), the percent weight loss can be attributed to the breaking of the alginate backbone with the fracture of glycosidic bonds which occurs at T^II^_max_ = 245.6 °C, with the loss of its abundant hydroxyl groups in the form of water [33]. In the highest temperature range (350–500 °C), a third mechanism is attributed to decarboxylation with the formation of calcium oxide and calcium hydroxide at 700° leading to a residue of about 32%. The thermogram profile of ALG@PDA (red curve) is similar to that of pure alginate, suggesting that PDA shells do not remarkably influence the thermal stability of alginate. In addition, in this case, three decomposition mechanisms were observed with a lower intensity and higher midpoint of thermal degradation compared to the ALG graph (Table 2). This behaviour indicates a higher thermal stability of PDA-coated alginate microparticles, confirmed by a higher carbonaceous residue of ~4% occurring at 700 °C compared to pure alginate.

Swelling properties of the samples were qualitatively analyzed to evaluate the effect of the PDA coating on alginate particles, suggesting some differences in terms of re-hydration due to the presence of the PDA coating (see supplementary data). After the dropping of a single bi-distilled water droplet, a swelling of particles was observed for all the analysed samples. In the case of ALG@PDA, the initial size of the particle was reached within 10 min, confirming an active role of the PDA coating on the fluid transport mechanism and re-hydration process. 

**Figure 7:**
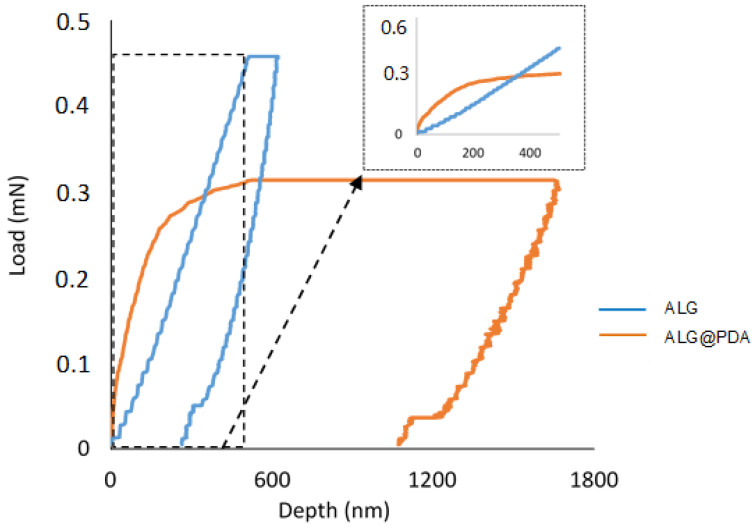
Nanoindentation tests: load vs. depth at fixed displacement (i.e., 500 nm) for ALG (blue) and ALG@PDA (orange). (For interpretation of the references to color in this figure legend, the reader is referred to the Web version of this article.).

In order to investigate the mechanical properties of the PDA layer on the alginate beads, force-controlled nanoindentation measurements were performed. It is well known that adhesion interactions exerted by cells onto the substrate fall in a sub micrometric/nanometric range of forces. Therefore, bulk measurement for an estimation of mechanical properties at higher size scales (i.e., micron or greater) may be not adequate, but more accurate measurements by the application of local nanoNewton (nN) forces at the sub microscale are strictly required. In this context, nanoindentation tests—typically used in material science to collect information for macroscopically homogeneous samples, such as polycrystalline solids, amorphous solids, and even nanocomposite structures [34,35,36,37]—were optimized to investigate PDA surface properties in core-shell architecture, overcoming some inherent difficulties in experimental set-up and data analysis. In particular, dry nanoparticles of alginate with (ALG@PDA) and without (ALG) coating were immobilized on a glass slice and indented to record the force vs. displacement curve, as reported in Figure 7. To disregard the presence of the surrounding glass slide, all the tests were performed at a very low indentation depth. The experimental nanoindentation tests (supplementary data, Figure S2) showed two main results: (a) the profile clearly indicated that ALG particles reveal a smooth depth curve until the maximum set penetration displacement with negligible variation of the slope, whereas in the case of ALG@PDA, a completely different trend is revealed with a rising curve highlighting the different stiffness interface encountered by the loading penetrator; and (b) the 60 sec holding segment at maximum reached force, resembling a creep loading mode of the nanoparticles and the level of displacement under constant load is very different between the two species. The creep displacement, in the case of core-shell nanoparticles, results 10 times higher (~1000 nm) compared with the corresponding value in the case of ALG (~100 nm).

According to the previous results, during the first loading stage, the interface stiffness calculated in the case of ALG@PDA—as a function of the penetration depth—confirmed the reinforcing role of the PDA shell surrounding the alginate core (Supplementary data—Figure S3). This result is also corroborated by the creep test performed using different loads (respectively, σ_max_ALG = 0.45 mN and σ_max_ALG@PDA = 0.30 mN) that confirm a significant difference in terms of resistance in the presence of the PDA coating. It is clearly remarked also by the force vs. depth curve recorded in the nanoindentation tests (Figure 7). It is clearly noticeable that the PDA shell reacts differently in respect to the alginate core and to the applied force profile for narrow displacement levels, thus ascribable to the crossing of the synthetized PDA shell with the surrounging alginate substrate, in agreement with data previously collected for similar substrates [16].

Starting from the experimental data on the mechanical response, in vitro tests were performed to investigate the interaction of the PDA shell with hMSC as a function of biomechanical properties.

**Figure 8:**
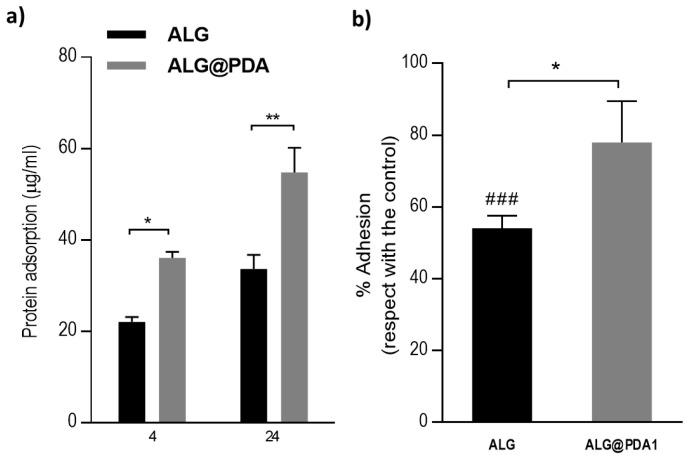
(**a**) Comparison of protein adsorption between ALG and ALG@PDA1 after 4 and 24 h. (**b**) Cell adhesion after 24 h of cell culture onto ALG and ALG@PDA1. Results are presented as percentage of cell adhesion of hBMSC respect to the TCP. # statistically significant difference against TCP (### *p* ≤ 0.001) * statistically significant difference between groups (* *p* ≤ 0.05; ** *p* ≤ 0.01).

It is well known that PDA coating concurs to improve the biocompatibility and hydrophilicity of the surrounding materials [28]. Moreover, PDA also identifies as an efficient spacer to bridge bioactive molecules and improve biological functionalities [38]. In this study, the effect of PDA coating on cell adhesion and proliferation of hMSCs was evaluated. In according with previous studies [39], it is demonstrated that PDA promotes protein adsorption on the surface, assuring a good adhesive response of cells. Figure 8a shows the amount of adsorbed protein of ALG and ALG@PDA. Results showed that the coating of PDA increased the protein adsorption after 4 h. The amount of adsorbed protein was larger on ALG@PDA1 microgels than on uncaoted ALG. The increased protein adsorption of PDA-coated microgels can be related to the interaction with amine or thiol groups through Shiff-base or Michael addition chemistry, that promote the adsorption of serum proteins, and improvement of cell adhesion [40]. The hMSCs adhesion after 24 h onto ALG and ALG@PDA at different concentrations is reported in Figure 8b. The percentage of cell adhesion with respect to the control—i.e., cell culture plate, TCP—is more than 60% in the case of PDA-coated microgels, differently to alginate microgels, confirming the higher ability of ALG@PDA to promote cell adhesion after 24 h with respect to the ALG. Moreover, ALG@PDA showed a comparable cell adhesion to the TCP control. These data underlined the role of catechol and amine groups that primarily contribute to the adhesive properties of PDA, thus counteracting the lack of adhesion sequences of alginate, in agreement with previous work [41].

**Figure 9:**
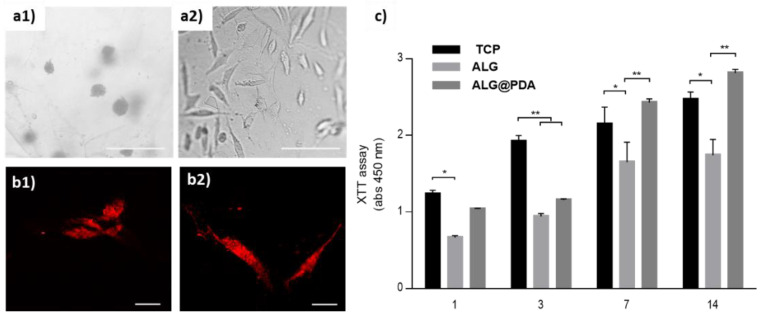
(**a**) Optical images of hMSCs after 3 days of culture onto the ALG (**a1**) and ALG@PDA microgels (**a2**). Scale bar: 200 μm. (**b**) Confocal images of hMSCs seeded onto ALG (**b1**) and ALG@PDA (**b2**) microgels (scale bar 20 μm). (**c**) Proliferation of hMSCs in ALG and ALG@PDA at 1, 3, 7 and 14 days. (Significant differences are indicated * *p* ≤ 0.05; ** *p* ≤ 0.01.)

This is confirmed by optical images of hMSCs after 3 days in culture (Figure 9a). ALG tends to present cells with a rounded shape, being indicative of weak interactions with alginate microgel, while ALG@PDA promotes a relevant spreading of cells along the surface, strictly due to an increase in protein adsoprtion attributed to the presence of PDA. These results are corroborated by confocal images that confirm a more prononced spreading of cells that follows the curvature of microgels, mainly in the presence of the PDA coating. In contrast, in hMSCs seeded onto ALG surfaces, the cells tend to maintain a rounded shape with a limited number of branchings (Figure 9b).

In addition, the promising use of core-shell micro-carriers for in vitro culture studies was proved in several studies. Indeed, their high surface area is potentially suitable for cells’ attachment, but a chemical functionalization is often required to induce a selective recognition of cells [42]. In this case, alginate is biocompatible and not cytotoxic, but a low cell affinity, high hydrophilicity, and high swelling ratio are fundamental constraints that limit cell proliferation at longer times [10].

Herein, it has been verified that the addition of a PDA coating can improve the interfacial properties—i.e., biochemical and biomechanical ones—able to support cell interactions over the integrin-based mechanisms. The effect of PDA coating on hMSCs proliferation was described by XTT assays. Overall, an increase in cell proliferation in the presence of PDA was observed for up to 14 days, for all the groups (Figure 9c). This result is in agreement with previous studies that confirm a good in vitro stability of PDA for long-term residence in culture media [43]. Indeed, PDA promotes a higher cell proliferation, with respect to the non-coated microgels after 3 days, progressively showing an increasing affinity until 14 days. This can be indirectly related to changes in swelling and mechanical properties of alginate microgels—namely, higher mechanical strength and lower water uptake—that are strictly determined by the PDA coating able to influence both chemical and physical properties of the surface hydrogels, thus improving the in vitro cell interface.

## 4. Conclusions

In this study, core-shell microgels were produced by a simple additive procedure based on the sequential use of electrodynamic atomization and an in situ precipitation reaction. This allowed the fabrication of ALG@PDA with core-shell architecture—i.e., core of alginate and a shell of PDA—mechanically robust and highly stable under biological conditions, better than alginate alone. An accurate optimization of chemical synthesis and atomization process conditions allowed the fine tuning of the final surface properties in terms of chemical and mechanical properties. Meanwhile, in vitro studies also confirmed an active role of the PDA coating on hMSC response in terms of protein adsorption, cell adhesion, and proliferation. All these results validate the idea that a PDA with peculiar biochemical and biomechanical cues concurs to generate bio-instructive interfaces that are better recognized by cells in vitro, with respect to alginate alone. Moreover, PDA macromolecules could be also used as spacers to selectively bind bioactive molecules, guaranteeing an efficient immobilization of growth factors suitable to specifically address, in perspective, cell fate in terms of cell differentiation. In the next future, core-shell microgels could be successfully used as advanced 3D in vitro models—with tailored features in terms of surface, fluid transport, and mechanical properties—in order to physically and chemically mimic the in vivo-like microenvironment towards a suitable approach to minimize the experimental use of animal testing in clinical trials.

## Figures and Tables

**Table 1:** Image analysis: evaluation of the average diameters.

DA (mg/mL)	Average Diameter (µm)	DA (mg/mL)
0	882.75 ± 31.70	0
0.5	875.08 ± 29.38	0.5

**Table 2:** Summary of TGA data for ALG and ALG@PDA.

Sample	T^I^max (°C)	T^II^max (°C)	T^III^max (°C)	Residue (%)
ALG	197.2 ± 0.3	245.6 ± 0.4	476.4 ± 0.4	35.4 ± 0.2
ALG@PDA	208.5 ± 0.4	255.4 ± 0.3	477.0 ± 0.3	31.8 ± 0.2

## Data Availability

All data that support the findings of this study are included within the article (and any supplementary files).

## Supplementary Materials

The following supporting information can be downloaded at: https://www.mdpi.com/article/10.3390/jfb14010002/s1, Figure S1. Optical images of (a) ALG and (b) ALG@PDA particles for qualitative analysis of swelling behavior. Scale bar 200 nm. Images show a swelling behavior in both particles. However, for ALG@PDA, the initial size of the particle was reached within 10 min, while ALG particles did not recover their initial size even after a longer time (1 h). This result confirmed an active role of the PDA coating on the mechanism of fluid transport and the re-hydration process. Figure S2. Nanoindentation tests: (a) force and (b) depth profile to achieve 500 nm indentation on ALG and ALG@PDA particles, respectively. Figure S2a reports the load profile applied to both samples up to a penetration depth of 500 nm. The first noticeable result is related to maximum force σmax (500 nm) to reach the final set penetration, which corresponds to ~0.45 mN and ~0.30 mN, respectively, for the solid alginate and the ALG@PDA particles. Figure S2b shows the recorded depth profiles following the imposed controlled force. Figure S3. Nanoindentation test on ALG@PDA at the same locations for two fixed penetrating values: 500 nm (black curve) and 2000 nm (green curves) along with bi-linearity of the path within the range 10–25 nm. Results reveal that the corresponding recorded path follows a knee-like profile within the referred range (i.e., 10–25 nm) likely due to the crossing of the synthetized PDA shell.

## References

[1] Kamata H., Li X., Chung U., Sakai T. (2015). Design of Hydrogels for Biomedical Applications. Adv. Healthc. Mater..

[2] Karg M., Pich A., Hellweg T., Hoare T., Lyon L.A., Crassous J.J., Suzuki D., Gumerov R.A., Schneider S., Potemkin I.I. (2019). Nanogels and Microgels: From Model Colloids to Applications, Recent Developments, and Future Trends. Langmuir.

[3] Guarino V., Gloria A., Raucci M.G., Ambrosio L. (2012). Hydrogel-based platforms for the regeneration of osteochondral tissue and intervertebral disc. Polymers.

[4] Guarino V., Altobelli R., Cirillo V., Cummaro A., Ambrosio L. (2015). Additive electrospraying: A route to process electrospun scaffolds for controlled molecular release. Polym. Adv. Technol..

[5] Guarino V., Altobelli R., Ambrosio L. (2016). Chitosan Microgels and Nanoparticles via Electrofluidodynamic Techniques for Biomedical Applications. Gels.

[6] Naqvi S.M., Vedicherla S., Gansau J., McIntyre T., Doherty M., Buckley C.T. (2016). Living Cell Factories—Electrosprayed Microcapsules and Microcarriers for Minimally Invasive Delivery. Adv. Mater..

[7] Guarino V., Caputo T., Calcagnile P., Altobelli R., Demitri C., Ambrosio L. (2018). Core/shell cellulose-based microspheres for oral administration of Ketoprofen Lysinate. J. Biomed. Mater. Res. Part B Appl. Biomater..

[8] Guarino V., Altobelli R., Caputo T., Ambrosio L., Caserta S., Calcagnile P., Demitri C. (2019). Mono- and Bi-Phasic Cellulose Acetate Micro-Vectors for Anti-Inflammatory Drug Delivery. Pharmaceutics.

[9] Zuppolini S., Maya I.C., Diodato L., Guarino V., Borriello A., Ambrosio L. (2020). Self-associating cellulose-graft-poly(ε-caprolactone) to design nanoparticles for drug release. Mater. Sci. Eng. C.

[10] Cruz-Maya I., Altobelli R., Marrese M., Guarino V. (2021). Design of alginate based micro-gels via electro fluid dynamics to construct microphysiological cell culture systems. Polym. Adv. Technol..

[11] Cavo M., Caria M., Pulsoni I., Beltrame F., Fato M., Scaglione S. (2018). A new cell-laden 3D Alginate-Matrigel hydrogel resembles human breast cancer cell malignant morphology, spread and invasion capability observed “in vivo”. Sci. Rep..

[12] Cruz-Maya I., Guarino V. (2022). 3D Scaffolds Fabrication via Bicomponent Microgels Assembly: Process Optimization and In Vitro Characterization. Micromachines.

[13] Liu Y., Nambu N.O., Taya M. (2017). Cell-laden microgel prepared using a biocompatible aqueous two-phase strategy. Biomed. Microdevices.

[14] Shahriari D., Koffler J., Lynam D.A., Tuszynski M.H., Sakamoto J.S. (2016). Characterizing the degradation of alginate hydrogel for use in multilumen scaffolds for spinal cord repair. J. Biomed. Mater. Res. Part A.

[15] Liu Y., Ai K., Lu L. (2014). Polydopamine and its derivative materials: Synthesis and promising applications in energy, environmental, and biomedical fields. Chem. Rev..

[16] Zotti A., Zuppolini S., Borriello A., Zarrelli M. (2020). Thermal and Mechanical Characterization of an Aeronautical Graded Epoxy Resin Loaded with Hybrid Nanoparticles. Nanomaterials.

[17] Maier G.P., Rapp M.V., Waite J.H., Israelachvili J.N., Butler A. (2015). Adaptive synergy between catechol and lysine promotes wet adhesion by surface salt displacement. Science.

[18] Godoy-Gallardo M., Portolés-Gil N., López-Periago A.M., Domingo C., Hosta-Rigau L. (2020). Multi-layered polydopamine coatings for the immobilization of growth factors onto highly-interconnected and bimodal PCL/HA-based scaffolds. Mater. Sci. Eng. C.

[19] Liu H., Li W., Wen W., Luo B., Liu M., Ding S., Zhou C. (2017). Mechanical properties and osteogenic activity of poly(l-lactide) fibrous membrane synergistically enhanced by chitosan nanofibers and polydopamine layer. Mater. Sci. Eng. C.

[20] Guarino V., Ambrosio L., Bellini D. (2009). Process for the Preparation of Microspheres Comprising Semisynthetic Polymers. International Patent.

[21] Cao H., Duan L., Zhang YCao J., Zhang K. (2021). Current hydrogel advances in physicochemical and biological response-driven biomedical application diversity. Signal Transduct. Target Ther..

[22] Ashimova A., Yegorov S., Negmetzhanov B., Hortelano G. (2019). Cell Encapsulation Within Alginate Microcapsules: Immunological Challenges and Outlook. Front. Bioeng. Biotechnol..

[23] Gepp M.M., Fischer B., Schulz A., Dobringer J., Gentile L., Vásquez J.A., Neubauer J.C., Zimmermann H. (2017). Bioactive surfaces from seaweed-derived alginates for the cultivation of human stem cells. J. Appl. Phycol..

[24] Wang K., Wang Z., Hu H., Gao C. (2022). Supramolecular microgels/microgel scaffolds for tissue repair and regeneration. Supramol. Mater..

[25] Altobelli R., Guarino V., Ambrosio L. (2016). Micro-and nanocarriers by electrofludodynamic technologies for cell and molecular therapies. Process Biochem..

[26] Manferdini C., Gabusi E., Saleh Y., Lenzi E., D’Atri G., Ricotti L., Lisignoli G. (2022). Mesenchymal Stromal Cells Laden in Hydrogels for Osteoarthritis Cartilage Regeneration: A Systematic Review from In Vitro Studies to Clinical Applications. Cells.

[27] Orlacchio R., Zuppolini S., Cruz-Maya I., Pragliola S., Borriello A., Guarino V., Fittipaldi R., Lettieri M., Venditto V. (2022). Polydopamine-Coated Poly-Lactic Acid Aerogels as Scaffolds for Tissue Engineering Applications. Molecules.

[28] Zuppolini S., Cruz-Maya I., Guarino V., Borriello A. (2020). Optimization of Polydopamine Coatings onto Poly–ε–Caprolactone Electrospun Fibers for the Fabrication of Bio-Electroconductive Interfaces. J. Funct. Biomater..

[29] Cheng W., Zeng X., Chen H., Li Z., Zeng W., Mei L., Zhao Y. (2019). Versatile Polydopamine Platforms: Synthesis and Promising Applications for Surface Modification and Advanced Nanomedicine. ACS Nano.

[30] Gibson C.T., Ridings C.R., Blok A.J., Shearer C.J., Andersson G.G., Ellis A. (2019). V Morphological changes of sintered polydopamine coatings. Surf. Topogr. Metrol. Prop..

[31] Zhang C., Gong L., Xiang L., Du Y., Hu W., Zeng H., Xu Z.-K. (2017). Deposition and Adhesion of Polydopamine on the Surfaces of Varying Wettability. ACS Appl. Mater. Interfaces.

[32] Rella S., Mazzotta E., Caroli A., De Luca M., Bucci C., Malitesta C. (2018). Investigation of polydopamine coatings by X-ray Photoelectron Spectroscopy as an effective tool for improving biomolecule conjugation. Appl. Surf. Sci..

[33] Kusuktham B., Prasertgul J., Srinun P. (2014). Morphology and Property of Calcium Silicate Encapsulated with Alginate Beads. Silicon.

[34] Sherstova T., Stokke B.T., Skallerud B., Maurstada G., Prot V.E. (2016). Nanoindentation and finite element modelling of chitosan–alginate multilayer coated hydrogels. Soft Matter.

[35] Baniasadi M., Minary-Jolandan M. (2015). Alginate-Collagen Fibril Composite Hydrogel. Materials.

[36] Díez-Pascual A.M., Gómez-Fatou M.A., Ania F., Flores A. (2015). Nanoindentation in polymer nanocomposites. Prog. Mater. Sci..

[37] Akhtar R., Draper E.R., Adams D.J., Hay J. (2018). Oscillatory nanoindentation of highly compliant hydrogels: A critical comparative analysis with rheometry. J. Mater. Res..

[38] Lee Y.B., Shin Y.M., Lee J.H., Jun I., Kang J.K., Park J.C., Shin H. (2012). Polydopamine-mediated immobilization of multiple bioactive molecules for the development of functional vascular graft materials. Biomaterials.

[39] Felgueiras H.P., Antunes J.C., Martins M.C.L., Barbosa M.A., Barbosa M.A., Martins M.C.L. (2018). Fundamentals of Protein and Cell Interactions in Biomaterials. Peptides and Proteins as Biomaterials for Tissue Regeneration and Repair.

[40] Lynge M.E., Schattling P., Städler B. (2015). Recent developments in poly(dopamine)-based coatings for biomedical applications. Nanomedicine.

[41] Kianersi S., Varjani A.A.A., Solouk A., Ai J., Lee B.P. (2020). Mussel-inspired polydopamine-coated silk fibroin as a promising biomaterial. Bioinspired Biomim. Nanobiomater..

[42] Smith D., Herman C., Razdan S., Abedin M.R., Van Stoecker W., Barua S. (2019). Microparticles for Suspension Culture of Mammalian Cells. ACS Appl. Bio Mater..

[43] Yan J., Wu R., Liao S., Jiang M., Qian Y. (2020). Applications of Polydopamine-Modified Scaffolds in the Peripheral Nerve Tissue Engineering. Front. Bioeng. Biotechnol..

