# Supplementary material for: Polydopamine-Coated Alginate Microgels: Process Optimization and In Vitro Validation
Source: J Funct Biomater. 2022 Dec 20;14(1):2. doi: 10.3390/jfb14010002 (PMC9865381; doi:10.3390/jfb14010002)
Supplement: Supplementary file 1 [file jfb-14-00002-s001.zip › jfb-2052096-supplementary.pdf]

Supporting information

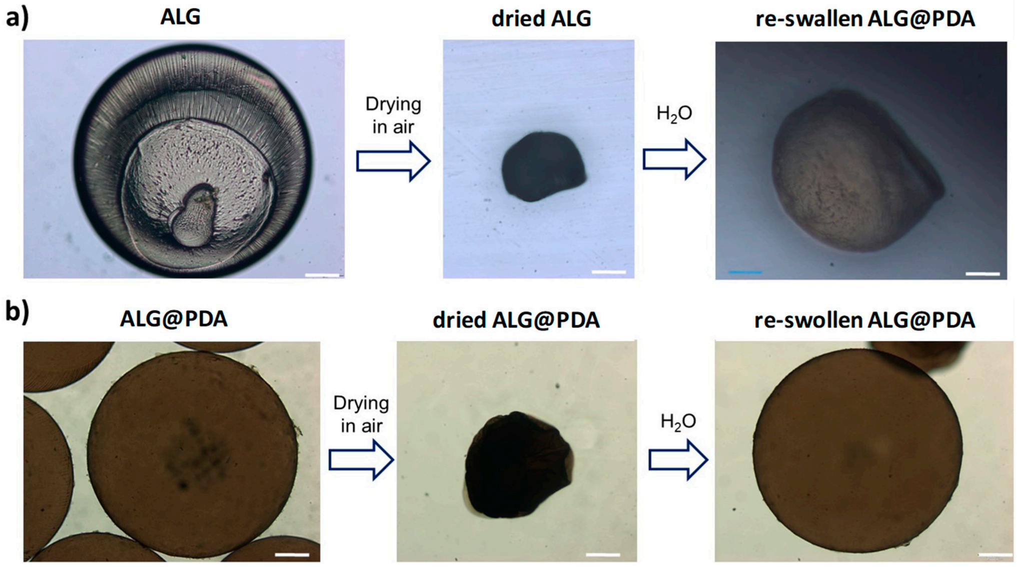

Figure S1. Optical images of a) ALG and b) ALG@PDA particles for qualitative analysis of swelling behaviour. Scale bar 200 nm. Images shows a swelling behavior in both particles. However, for ALG@PDA the initial size of the particle was reached within 10 min, while ALG particles did not recover its initial size even after longer time (1 hour). This result confirmed an active role of PDA coating on the mechanism of fluid transport and re-hydration process.

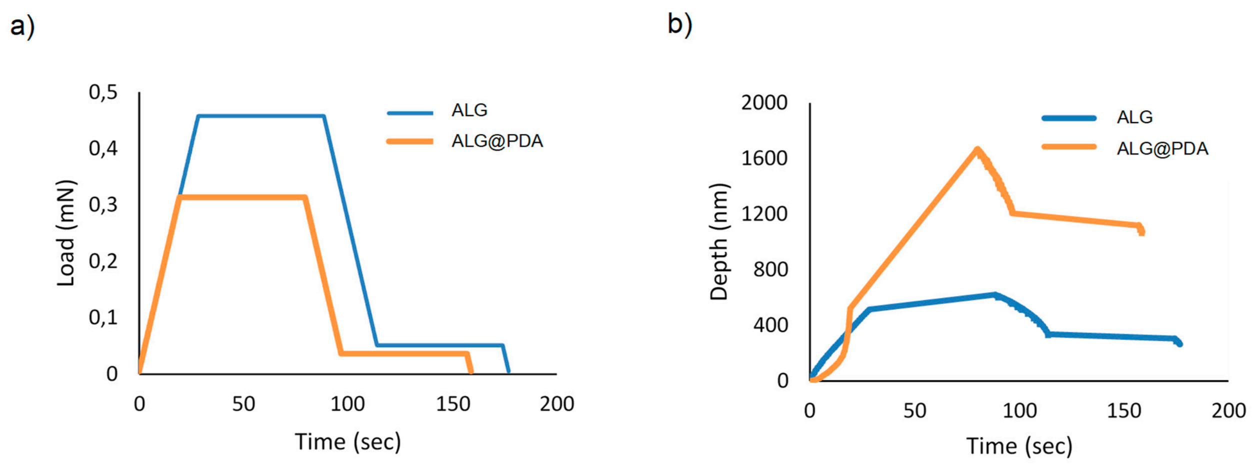

Figure S2. Nanoindentation tests: a) force and b) depth profile to achieve 500 nm indentation on ALG and ALG@PDA particles, respectively. Figure S2.a reports the load profile applied to both samples up to a penetration depth of 500 nm. The first noticeable result is related to maximum force σ_{max} (500 nm) to reach the final set penetration, which corresponds to ~0.45 mN and ~0.30 mN respectively for the solid alginate and the ALG@PDA particles. Figure S2.b. shows the recorded depth profiles following the imposed controlled force.

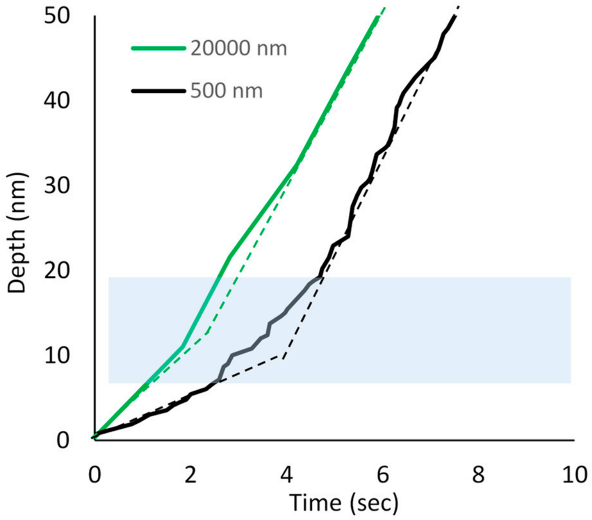

Figure S3. Nanoindentation test on ALG@PDA at same locations for two fixed penetrating values: 500 nm (black curve) and 2000 nm (green curves) along with bi-linearity of the path within the range 10 - 25 nm. Results reveal that the corresponding recorded path follows a knee-like profile within the referred range (i.e., 10 nm - 25 nm) likely due to the crossing of the synthetized PDA shell.
